# Stimulation of translation by human Unr requires cold shock domains 2 and 4, and correlates with poly(A) binding protein interaction

**DOI:** 10.1038/srep22461

**Published:** 2016-03-03

**Authors:** Swagat Ray, Emma C. Anderson

**Affiliations:** 1 School of Life Sciences, University of Warwick, Coventry, CV4 7AL, UK

## Abstract

The RNA binding protein Unr, which contains five cold shock domains, has several specific roles in post-transcriptional control of gene expression. It can act as an activator or inhibitor of translation initiation, promote mRNA turnover, or stabilise mRNA. Its role depends on the mRNA and other proteins to which it binds, which includes cytoplasmic poly(A) binding protein 1 (PABP1). Since PABP1 binds to all polyadenylated mRNAs, and is involved in translation initiation by interaction with eukaryotic translation initiation factor 4G (eIF4G), we investigated whether Unr has a general role in translational control. We found that Unr strongly stimulates translation *in vitro*, and mutation of cold shock domains 2 or 4 inhibited its translation activity. The ability of Unr and its mutants to stimulate translation correlated with its ability to bind RNA, and to interact with PABP1. We found that Unr stimulated the binding of PABP1 to mRNA, and that Unr was required for the stable interaction of PABP1 and eIF4G in cells. siRNA-mediated knockdown of Unr reduced the overall level of cellular translation in cells, as well as that of cap-dependent and IRES-dependent reporters. These data describe a novel role for Unr in regulating cellular gene expression.

Unr (also known as CSDE1) is a cellular RNA-binding protein with five cold shock domains, and a preference for binding purine-rich RNA sequences^1^. It was first discovered to function in translational control as a factor required for translation from the human rhinovirus type 2 (HRV-2) internal ribosome entry site (IRES)^2^. The requirement of all five cold shock domains of Unr^3^, and binding sites in different subdomains of the IRES^4^, suggested that Unr acts as an RNA chaperone to maintain the active HRV-2 IRES structure. Unr has since been shown to function as a stimulator of cap-independent translation of poliovirus type 1 RNA^5^, apoptotic protease activating factor 1 mRNA^6^, and the p58 isoform of CDK11 mRNA^7^. Unr can also have a negative effect on translation initiation; binding to the IRES in its own transcript represses its translation^8^, Unr is part of a translation repressor complex that binds to the 5′ UTR of poly(A) binding protein 1 (PABP1) mRNA^9^, and in *Drosophila*, Unr is part of a repressor complex that forms on the 3′ UTR of the male-specific lethal 2 (msl2) mRNA^10^,^11^, inhibiting ribosome recruitment^12^. There are also examples of Unr regulating mRNA stability: in the case of c-fos mRNA, Unr is part of a complex that promotes translationally-coupled mRNA turnover^13^, whereas Unr is part of a stabilising complex on the parathyroid hormone mRNA^14^. In these varied roles, Unr functions in concert with other protein partners; one such protein is PABP1, with which Unr interacts on PABP1 mRNA^9^, c-fos RNA^13^, and on msl2 mRNA^12^ in *Drosophila*. In binding polyadenylated mRNAs, PABP1 has universal roles in gene expression, mediating both mRNA stability and translation initiation, the latter through its interaction with eukaryotic initiation factor 4G (eIF4G)^15^, which can bring the 5′ and 3′ ends of an mRNA together^16^. Therefore, while Unr has been shown to have specific functions in regulating gene expression of the above-mentioned mRNAs, Unr may have a wider role in gene expression given its RNA binding and PABP1 binding activities. We have investigated the role of Unr in regulating cellular translation and found that Unr stimulates translation *in vitro* and in cells, and that this correlates with its ability to interact with PABP1.

## Results

### Unr stimulates translation *in vitro*

**Figure 1:**
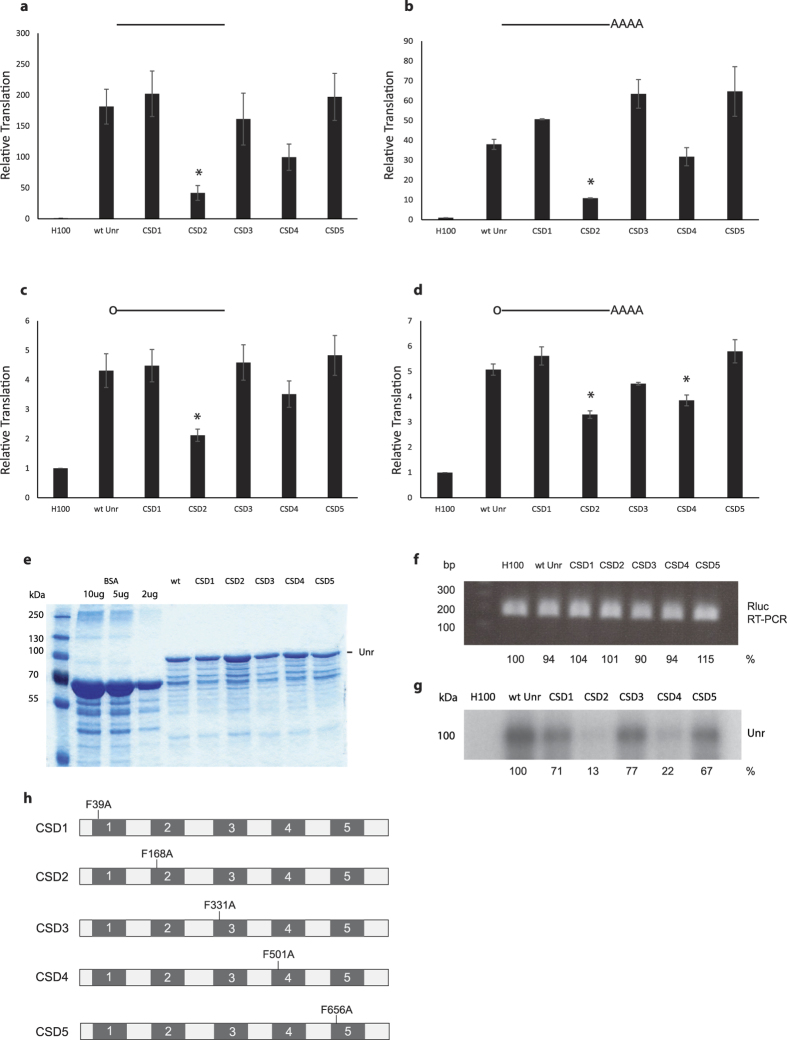
Unr stimulates translation *in vitro*. **(a–d)** *In vitro* translation of uncapped non-polyadenylated **(a)**, uncapped polyadenylated **(b)**, capped non-polyadenylated **(c)** or capped polyadenylated **(d)** *Renilla* luciferase mRNA supplemented with wild type (wt) Unr or CSD mutants of Unr, relative to the buffer control (H100). The presence of a cap (O) and/or poly(A) tail (AAAA) on the mRNA (horizontal line) is indicated above each graph. Unr was in 3.7 fold molar excess over mRNA. The graphs show the mean of three independent experiments, error bars represent the standard deviation. An asterisk represents a significant difference (p < 0.05) to wt Unr. **(e)** Coomassie stained polyacrylamide gel showing purified recombinant wt and mutant Unr proteins, alongside BSA for quantification purposes. **(f)** RT-PCR of capped polyadenylated *Renilla* luciferase RNA isolated from *in vitro* translation reactions, supplemented as above. PCR products were quantified using ImageJ analysis of the agarose gel image; the values are shown beneath the gel image, relative to the buffer control. **(g)** UV crosslinking of wt Unr and CSD mutants of Unr to capped polyadenylated ^32^P-UTP-labelled *Renilla* luciferase mRNA. RNA-binding of Unr and CSD mutant proteins was quantified using ImageJ analysis of the autoradiograph; the values are shown beneath the autoradiograph, relative to wt Unr. **(h)** Schematic of the CSD mutants of Unr. The amino acid position of F to A point mutations in each mutant is indicated. Amino acid numbering according to Unr protein isoform 2^23^, which was the recombinant form of Unr used in this study.

Rabbit reticulocyte lysate contains very little Unr, and so the effect of Unr on translation *in vitro* can be observed by supplementing the lysate with recombinant protein^2^. Wild type (wt) Unr increased the translation of an uncapped non-polyadenylated *Renilla* luciferase mRNA that contains a short unstructured 5′ untranslated region almost 200-fold (Fig. 1a). Wild type Unr also stimulated translation of uncapped polyadenylated mRNA (Fig. 1b, 40-fold), capped non-polyadenylated mRNA (Fig. 1c, 4-fold) and capped polyadenylated mRNA (Fig. 1d, 5-fold). The differences in the fold increase in translation reflects the fact that the uncapped mRNAs had a lower basal level of translation and were stimulated more than the already well-translated capped mRNAs. The addition of mutants of Unr, each containing a point mutation in the RNP-1 motif of a single cold shock domain (CSD)^3^, showed that CSDs 2 and 4 were required for translation stimulation activity but that CSDs 1, 3 and 5 were not. The same pattern of results was seen with all four mRNAs (Fig. 1a–d), although the difference between the translation activity of wt Unr and the CSD2 mutant was significant in each case, whereas the difference between wt Unr and the CSD4 mutant was only significant on capped polyadenylated mRNA. Interestingly, the effect of mutating CSD2 was much greater on uncapped than capped mRNAs. The recombinant Unr proteins used in these assays were of equivalent quality (Fig. 1e), and RT-PCR of the *Renilla* luciferase mRNA at the end of a typical *in vitro* translation experiment showed very little difference in mRNA levels (Fig. 1f). This confirms that the effect of Unr was on translation rather than mRNA stability. We next tested the ability of wt Unr and the mutants to bind to ^32^P-UTP-labelled *Renilla* luciferase mRNA in a UV crosslinking assay; following incubation of RNA and protein, and UV irradiation, the sample is digested with a mixture of RNases. This leaves the protein radiolabelled only with nucleotides to which it was directly bound. CSD mutants 2 and 4 were severely impaired in their ability to bind the *Renilla* luciferase mRNA, with only 13 and 22% of the binding activity of wt Unr, whereas CSD mutants 1, 3 and 5 were only modestly impaired (71, 77 and 67% binding activity relative to wt Unr, respectively). We observed the same pattern of results in UV crosslinking assays whether the mRNA was capped or uncapped. These results show that there is a correlation between the ability of Unr mutants to bind the mRNA and to stimulate translation from it, but the fact that CSD mutants with very little RNA-binding activity maintain some translation activity suggests that RNA binding may not be the only factor that governs Unr’s translation activity.

### Unr interacts with PABP1 *in vitro* and stimulates mRNA binding by PABP1

**Figure 2:**
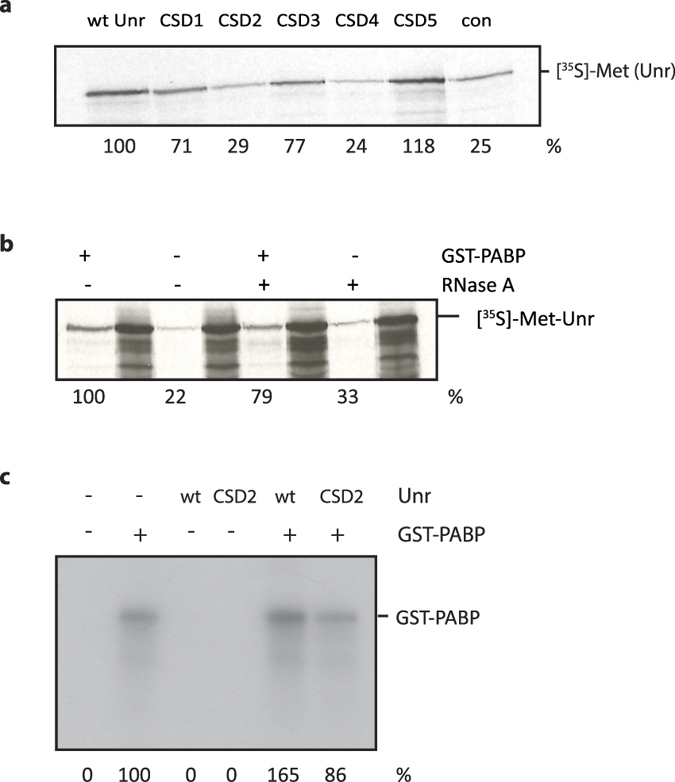
Unr interacts with PABP1 *in vitro.* **(a)** GST-pull down of ^35^S-labelled wt Unr and CSD mutants with GST-PABP1, or with GST as a negative control (con). Unr pull down was quantified using ImageJ analysis of the autoradiograph, normalised against inputs; the values are shown beneath the autoradiograph, relative to wt Unr binding to GST-PABP1. **(b)** GST-pull down of ^35^S-labelled wt Unr with or without GST-PABP1, in the presence or absence of RNase A. The inputs are shown to the right of each pull down and are indicated by asterisks. Unr pull down was quantified using ImageJ analysis of the autoradiograph; the values are shown beneath the autoradiograph, relative to Unr binding to GST-PABP1 in the absence of RNase A. **(c)** UV crosslinking of GST-PABP1, wt Unr, CSD mutant 2, alone and in combination, to uncapped, non-polyadenylated ^32^P-ATP-labelled *Renilla* luciferase mRNA. RNA-binding of GST-PABP1 or Unr was quantified using ImageJ analysis of the autoradiograph; the values are shown beneath the autoradiograph, relative to GST-PABP1 alone. Experiments were carried out on three independent occasions and representative autoradiographs are shown.

We tested the ability of *in vitro* translated wt Unr and the CSD mutants to bind to GST-PABP1 in GST pull down assays; CSD mutants 2 and 4 were also most impaired in their ability to bind PABP1 (Fig. 2a, 29 and 24% binding activity, respectively). This was similar to the level of interaction that was seen between wt Unr and GST (Fig. 2a, right hand lane). Given the similar pattern of binding of the Unr mutants to RNA and PABP1, we examined whether the interaction between Unr and PABP1 was RNA-dependent. Addition of RNase A to the pull down only moderately reduced the interaction to 79% relative to no RNase A (Fig. 2b), suggesting that the interaction between wt Unr and PABP1 is not primarily mediated by RNA. We next investigated whether Unr modified the RNA-binding activity of PABP1. A UV crosslinking assay was performed with uncapped, non-polyadenylated ^32^P-ATP-labelled *Renilla* luciferase mRNA, to which GST-PABP1 could be seen to bind (Fig. 2c). Although the binding of Unr to the *Renilla* luciferase mRNA can be easily detected when the mRNA is labelled with ^32^P-UTP (Fig. 1g), it is not detected when the mRNA is labelled with ^32^P-ATP (Fig. 2c). This was surprising given that Unr is reported to bind purine-rich sequences, but it did enable us to test the effect of Unr on mRNA binding by PABP1 without detecting confounding bands on the autoradiograph. We found that the presence of wt Unr, but not CSD mutant 2, increased the binding of GST-PABP1 to the mRNA (Fig. 2c) in this assay. This could reflect increased recruitment of PABP1 to the mRNA in the presence of Unr, or the formation of a Unr-PABP1-mRNA complex that is more stable than PABP1-mRNA alone. Since these experiments were carried out using non-polyadenylated mRNA, this suggests that Unr is able to stimulate the binding of PABP1 to mRNA at positions other than the poly(A) tail.

### Unr increases the PABP1-eIF4G interaction in cells

**Figure 3:**
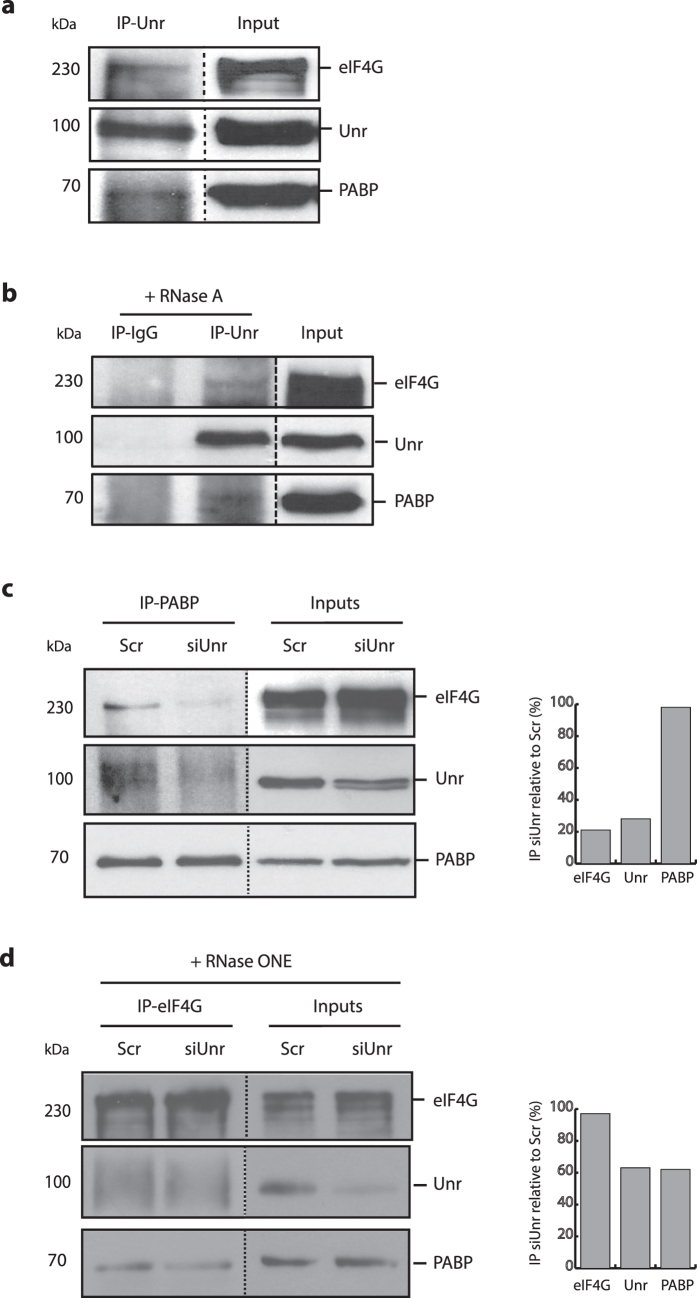
Unr increases the PABP1-eIF4G interaction in cells. **(a)** Immunoprecipitation (IP) from untreated HeLa cells with anti-Unr antiserum. **(b)** IP from untreated HeLa cells with anti-Unr antiserum or IgG control in the presence of RNase A. **(c)** IP from HeLa cells that had been pretreated with siRNA against Unr (siUnr) or a negative control siRNA (Scr), with anti-PABP1 antiserum. **(d)** IP from HeLa cells that had been pretreated with siRNA against Unr (siUnr) or a negative control siRNA (Scr), with anti-eIF4G antiserum in the presence of RNase ONE. For **(a–d)**, input lysates and IP samples were run on the same gel, blotted onto nitrocellulose and the membrane cut to allow probing with antibodies against eIF4G (top panels), Unr (middle panels) or PABP1 (lower panels). Images separated by dotted lines represent sections of the same membrane. For **(c**,**d)**, the percentage pull down of eIF4G, Unr and PABP1 from cells treated with Unr siRNA relative to control siRNA was quantified using ImageJ analysis, and is shown to the right of the western blots.

We used co-immunoprecipitation (co-IP) assays to investigate the interaction between endogenous Unr and PABP1 within the cellular environment. Although such experiments are technically more challenging than detecting interactions between overexpressed proteins, they more closely replicate physiological interactions. Indeed, we have found that overexpression of Unr causes cell stress, and changes to the subcellular distribution of Unr (Ray *et al.* manuscript in preparation). Endogenous PABP1 and eIF4G were both detected in western blots of Unr IPs, indicating that they interact with Unr within the cell (Fig. 3a). A similar result was obtained in the presence of RNase A (Fig. 3b), showing that the interaction between Unr, PABP1 and eIF4G proteins is not RNA-dependent. Also, IP with a non-specific IgG did not precipitate Unr, PABP1 or eIF4G (Fig. 3b).

To investigate whether Unr is required for complex formation between PABP1 and eIF4G, we used siRNA against Unr to reduce its expression followed by co-IP of proteins with PABP1. Western blotting of the input lysates showed that Unr expression was substantially reduced by the Unr-specific siRNA whereas PABP1 and eIF4G levels were unaffected (Fig. 3c, right hand lanes). At this low level of Unr, two isoforms that migrate very closely can be seen. IP of PABP1 pulled down both Unr and eIF4G from the negative control siRNA treated cells (Scr). Depletion of Unr led, as expected, to less Unr being pulled down by co-IP with PABP1, but importantly, less eIF4G was also detected (Fig. 3c, top panel, and quantified, right), suggesting that the eIF4G interaction with PABP1 is indeed enhanced by Unr. To extend these findings, we looked at this interaction in reverse, testing for co-IP with eIF4G, this time in the presence of RNase ONE to disrupt any RNA-mediated interactions. As before, Unr expression was substantially reduced by specific siRNA (Fig. 3d, right hand lanes). In the presence of Unr, IP of eIF4G pulled down PABP1 as expected, but it appeared that less Unr was precipitated by eIF4G IP than by PABP1 IP. This was not due to RNase ONE treatment of the lysate, as we saw no difference in IP between RNase treated and non-treated lysates (data not shown). In agreement with the result of co-IP via PABP1 (above), depletion of Unr reduced PABP1 co-IP with eIF4G (Fig. 3d, bottom panel, quantified, right). Together, these data fit a model in which Unr binds PABP1 directly, and eIF4G indirectly, via PABP1, with Unr stabilising the interaction between PABP1 and eIF4G.

### Unr stimulates translation in cells

**Figure 4:**
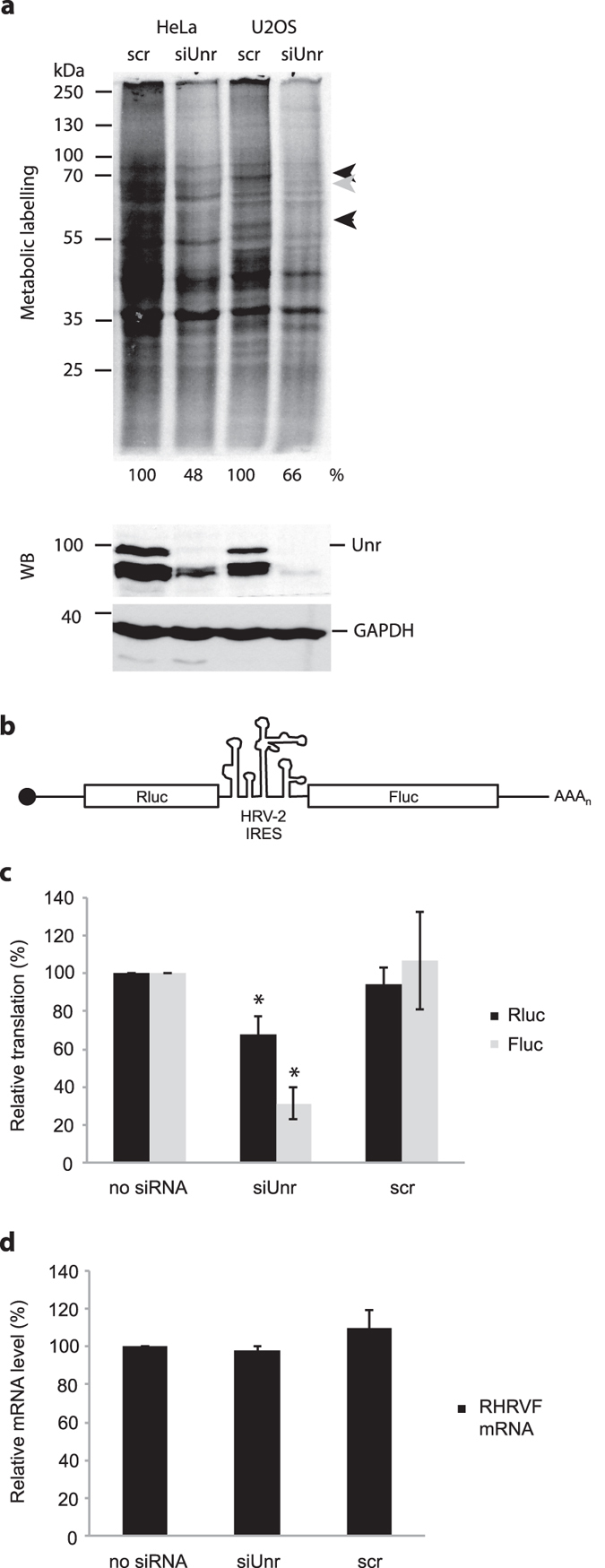
Unr stimulates translation in cells. **(a)** Metabolic labelling (upper panel) and western blotting for Unr and GAPDH (lower panels) of HeLa and U2OS cells that had been pretreated with siRNA against Unr (siUnr) or a negative control siRNA (Scr). ^35^S-methionine incorporation into nascent proteins was quantified using ImageJ analysis of the autoradiograph; the values are shown beneath the autoradiograph, relative to the negative control siRNA-treated samples for each cell type. Grey and black arrowheads indicate bands whose intensity increases or decreases, respectively, between negative control siRNA-treated samples and Unr siRNA-treated samples. This experiment was carried out on three independent occasions and a representative autoradiograph is shown. **(b)** Schematic of capped and polyadenylated dicistronic RHRVF mRNA. The upstream *Renilla* luciferase (Rluc) cistron is translated in a cap-dependent manner. Translation of the downstream firefly luciferase (Fluc) depends on the activity of the intercistronic HRV-2 IRES. **(c)** Relative translation of Rluc and Fluc from RHRVF mRNA 6 hours post-transfection of cells that had been pretreated with no siRNA, siRNA against Unr (siUnr) or a negative control siRNA (scr). The graph shows the means of three independent experiments and error bars represent the standard deviation. An asterisk represents a significant difference (p < 0.05) to the no siRNA sample. **(d)** Relative level of ^32^P-labelled dicistronic RHRVF mRNA 6 hours post-transfection of cells that had been pretreated with no siRNA, siRNA against Unr (siUnr) or a negative control siRNA (scr), quantified by scintillation counter. The graph shows the means of two independent experiments carried out in duplicate and error bars represent the standard deviation.

The effect of Unr on endogenous translation in cells was tested by treating cells for 48 hours with siRNA against Unr, or a negative control siRNA, followed by metabolic labelling of cells with ^35^S-methionine for 30 minutes. This experiment was carried out both in HeLa and U2OS (human osteosarcoma) cells. Western blotting of the cell lysates showed that a good level of knockdown of Unr (all isoforms) was achieved whereas the GAPDH control was unaffected (Fig. 4a, lower panels). In both HeLa and U2OS cells, the overall level of translation, as measured by ^35^S-methionine incorporation, was reduced in cells treated with siRNA against Unr (Fig. 4a, 48% and 66% of the level in negative control siRNA-treated cells, respectively). While it is difficult to distinguish the effect of Unr knockdown on the synthesis of most individual proteins (especially lower abundance proteins), the synthesis of a few of the high abundance proteins was strikingly reduced in the Unr knockdown cells (indicated by black arrowheads in Fig. 4a). On the other hand, a few proteins were synthesised to a higher level in the Unr knockdown cells (such as that indicated by a grey arrowhead in Fig. 4a), showing that Unr can also act as a repressor of translation of certain mRNAs.

We also tested the effect of siRNA-mediated Unr knockdown on the translation of a capped and polyadenylated dicistronic reporter RNA (RHRVF^6^, Fig. 4b) in which the upstream *Renilla* luciferase open reading frame is translated in a cap-dependent manner, and translation of the downstream firefly luciferase open reading frame is mediated by the human rhinovirus 2 IRES, which is known to require Unr for activity^2^. The dicistronic mRNA was used to transfect HeLa cells 48 hours after siRNA transfection, and cells were harvested 6 hours post-transfection. (It is not possible to similarly test uncapped mRNAs in cells due to their instability). There was a significant difference in translation of both cistrons between untreated and Unr siRNA treated cells. Translation of the HRV-2 IRES-dependent firefly luciferase (Fluc) was reduced by 70%, and translation of the cap-dependent Renilla luciferase (Rluc) was reduced by 33% in cells treated with Unr siRNA (Fig. 4c). The negative control siRNA had no effect on translation. To check that the reduction in luciferase synthesis was not due to a reduction in mRNA stability, the experiment was repeated using ^32^P-UTP-labelled dicistronic reporter mRNA. Upon harvesting the cells, total RNA was isolated and ^32^P-labelled mRNA quantified in a scintillation counter. There was no difference in the level of reporter mRNA between cells treated with no siRNA, siRNA against Unr, or a negative control siRNA (Fig. 4d). These experiments suggest that Unr is a general regulator of mammalian cell translation.

## Discussion

Unr has previously been shown to have a number of specific roles in post-transcriptional regulation of gene expression on specific mRNAs. However, a number of lines of indirect evidence suggest that Unr may have a wider role in cellular translation beyond the specific mRNAs previously described. The UTR database (http://utrdb.ba.itb.cnr.it) shows almost 3000 human mRNAs containing putative Unr binding sites in their 5′ or 3′ UTRs, based on RNA sequences identified by SELEX (systematic evolution of ligands by exponential enrichment) as Unr binding sequences^1^; this does not include mRNAs which may contain Unr binding sites within their coding region (such as c-fos mRNA^13^). A large subset of mRNAs isolated from HeLa cytoplasmic extract bound to a nickel agarose column pre-bound with histidine-tagged Unr (E. C. Anderson and R. J. Jackson, unpublished data), and we have recently identified approximately 4000 transcripts from HeLa cell lysate that were specifically pulled down by Unr in RIP-seq experiments (S. Ray, P. Ó. Catnaigh, N. Dyer, S. Ott and E. C. Anderson, unpublished data). A similar study in *Drosophila* identified more than 2000 mRNAs bound to Unr^17^. In this study we have demonstrated that human Unr has translation stimulation activity in cells beyond its role in IRES-dependent translation initiation.

*In vitro* translation experiments using recombinant Unr protein and mutants showed that the translation stimulation activity of Unr depends on CSDs 2 and 4 within its five domain structure. This is a different pattern to the activity of the Unr mutants on the HRV-2 IRES, in which mutation of any of the five CSDs inhibited both the ability of Unr to bind to the IRES efficiently and Unr’s activity in stimulating translation^3^ (although the CSD2 and 4 mutants did have the lowest HRV-2 RNA-binding activity of the five mutants). The dependence on CSDs 2 and 4 also contrasts with *Drosophila* Unr, whose role in inhibiting translation of msl2 mRNA depends on its glutamine-rich N terminal region (unique to *Drosophila* Unr) and CSDs 1 and 2, with the msl2 binding determinant mapped to CSD1^18^. While Unr’s role in HRV-2 IRES translation is likely to be the maintenance of an RNA structure required for ribosome recruitment, we suggest that Unr is likely to have a different role in the translation of less structured mRNAs that depends on its protein-binding activity. As has been reported in the case of the protein complexes that form on c-fos^19^ and PABP1^9^ mRNA, we found that Unr interacted directly with PABP1 in both *in vitro* and *in vivo* assays. Interestingly, CSDs 2 and 4 of Unr are required both for binding mRNA and for interacting with PABP1, although the interaction with PABP1 was only partially RNA-dependent. *In vivo,* we showed that Unr forms a complex with PABP1 and eIF4G and that knocking down Unr reduces the interaction between PABP1 and eIF4G. The interaction between eIF4G and PABP1 is crucial for efficient translation initiation^20^ and the ability of Unr to increase or stabilise this interaction, coupled with its ability to increase or stabilise PABP1 interaction with mRNA, could explain why Unr stimulates translation. This data could also explain why Unr stimulates translation from uncapped mRNA more efficiently than capped mRNA; the presence of Unr and PABP1 bound to an mRNA may substitute for the 5′ cap in recruiting eIF4G, and thus the 43S preinitiation complex, to the mRNA. The particularly deleterious effect of mutating CSD2 on translation of uncapped mRNAs suggests that this domain of Unr may be particularly important for such a role.

The results presented here suggest that Unr is a protein that can regulate both cap-dependent and cap-independent translation in human cells, and that it achieves this at least in part through its interaction with PABP1. It is clear that Unr is not essential for protein synthesis to occur – almost complete knockdown of Unr expression reduced total cellular translation by between one third and one half – but the activity of Unr can influence the rate of protein synthesis, and therefore is likely to affect the ability of the cell to respond to changing metabolic demands. There was also variation in the extent to which translation of individual mRNAs was reduced by Unr knockdown, suggesting that Unr could have greater impact on the functioning of some cellular pathways than others. Finally, as Unr can also repress the translation of certain mRNAs, it will be interesting to discover how Unr is partitioned to different mRNPs to regulate the proteome.

## Methods

### Recombinant protein production

Histidine-tagged Unr (NP_009089) and Unr CSD mutants, which have been described previously^3^, were overexpressed in *E. coli* BL21(DE3) cells from pET21d plasmids and purified with Ni^2+^-NTA agarose (Qiagen) according to the manufacturer’s recommendation. Eluted proteins were dialysed against H100 buffer (20 mM HEPES-KOH pH 7.5, 100 mM KCl, 2 mM DTT). GST-tagged PABP1 was overexpressed in *E. coli* BL21(DE3) cells from pGEX2T (a kind gift from O. de Melo Neto^21^), purified with glutathione Sepharose 4B (GE Healthcare Life Sciences) according to the manufacturer’s recommendation, and dialysed against H100 buffer.

### *In vitro* transcription and translation

*Renilla* luciferase mRNA was transcribed from pRL-CMV (Promega) linearised with Xba1. Dicistronic RHRVF mRNA was transcribed from pRHRVF^6^ linearised with BamH1. *In vitro* transcription for *in vitro* translation and UV crosslinking assays was carried out using a T7 maxiscript kit (Ambion) with or without cap analogue to generate capped or uncapped mRNA, followed by use of a polyadenylation kit (Ambion) if polyadenylated mRNA was required. Generation of capped and polyadenylated RHRVF mRNA for transfection into mammalian cells was carried out using a T7 mMESSAGE mMACHINE and polyadenylation kit (Ambion). *In vitro* translation assays were carried out in Flexi rabbit reticulocyte lysate (Promega) programmed with 20 ng/μl mRNA, with the addition of 0.5 mM MgCl_2_ and 60 mM KCl. The reactions were supplemented with 20 ng/μl recombinant Unr, Unr mutants, or H100 buffer as a control. Unr was in 3.7 fold molar excess over mRNA. Luciferase assays were carried out using the *Renilla-*Glo or dual luciferase assay systems (Promega).

### RT-PCR

Total RNA was isolated from completed *in vitro* translation reactions using an RNeasy mini-kit (Qiagen). cDNA was generated using a high capacity RNA-cDNA kit (Applied Biosystems) according to the manufacturer’s instructions, and used as a template for PCR. Primers used were: Rluc F 5′-ACGGATGATAACTGGTCCGC-3′ and Rluc R 5′-TAATACACCGCGCTACTGGC-3′. PCR reactions were run on a 1.8% TBE-agarose gel containing ethidium bromide alongside 100 bp DNA ladder (NEB) and visualised under UV light using a Gel Doc XR (BioRad).

### UV crosslinking

High specific activity ^32^P-labelled *Renilla* luciferase mRNAs were generated using the T7 maxiscript kit with 50 μCi ^32^P-UTP or ^32^P-ATP according to the manufacturer’s recommendation. UV crosslinking reactions were carried out as previously described^22^, but with 20 ng/μl recombinant Unr, Unr mutant proteins, or GST-PABP1 (as indicated in figure legends) and 60 mM KCl.

### GST pull down assays

25 μl of glutathione Sepharose 4B (GE Healthcare Life Sciences) was washed with 200 μl pull-down buffer (50 mM Tris pH 7.5, 100 mM KCl, 0.5 mM EDTA, 0.1% NP40, 1 mM DTT, 1 mM benzamidine). 2 μg recombinant GST-PABP1 was bound to the washed beads in 200 μl pulldown buffer by rotating the beads for 1 hr at 4 °C. Excess protein was washed off with 200 μl pull-down buffer. Unr and Unr CSD mutants were *in vitro* transcribed/translated from pET21d plasmids using TnT^®^ Quick Coupled Transcription/Translation System (Promega) supplemented with radiolabelled [^35^S] methionine, according to manufacturer’s protocol. Where indicated, 0.1 mg/ml RNase A was added to the TnT reaction following transcription/translation, and incubated for a further 30 min at 30 °C prior to pull down. 10 μl TnT reaction was added to GST-PABP1 bound beads and rotated for 1 hr at 4 °C. Beads were then washed twice with 200 μl pull-down buffer, boiled in SDS protein sample buffer and run on a 10% SDS-polyacrylamide gel. The gel was dried, exposed to BioMax MR film (Kodak) and [^35^S]-Unr bands were detected by autoradiography.

### Tissue culture and transfections

HeLa cells were cultured in DMEM plus 10% FBS (heat inactivated) while U2OS cells were cultured in McCoy’s 5A medium plus 10% FBS (heat inactivated) at 37 °C/5% CO_2_. For transfections, cells were grown in 6-well tissue culture plates, transfected with either 1 μg/well capped and polyadenylated RHRVF mRNA (unlabelled or trace-labelled with ^32^P-UTP), or 20 nM siRNA using Lipofectamine 2000 (Life Technologies). The negative control siRNA was *Silencer* negative control # 2 (Life Technologies); siRNA against unr was *Silencer* pre-designed siRNA to human CSDE1 (Life Technologies; ID 122624). Cells were harvested in 1× Passive Lysis Buffer (Promega) 6 hours (mRNA) or 48 hours (siRNA) post-transfection. Luciferase assays were carried out using a dual luciferase assay kit (Promega). To assess mRNA stability following transfection of HeLa cells with ^32^P-UTP-labelled RHRVF mRNA for 6 hours, total RNA was isolated using an RNeasy mini kit (Qiagen), and ^32^P was quantified in a Tri-Carb scintillation counter with QuantaSmart software (PerkinElmer).

### Immunoprecipitation and western blotting

Cells grown in 6-well tissue culture plates were collected to generate 100–200 μg of total protein per IP. Cells were pelleted by centrifugation (1000 × g) for 10 min at 4 °C, washing several times with 10 ml of ice cold phosphate buffered saline (PBS). Final pellet was resuspended with an approximately equal volume of polysome lysis buffer (PLB) [100 mM KCl, 5 mM MgCl_2_, 10 mM HEPES (pH 7.0), 0.5% NP40,1 mM DTT, protease inhibitor cocktail (Roche)]. The lysate was allowed to stand for 5 min on ice and then stored at −80 °C. 30 μl of Dynabeads^®^ Protein A (Life Technologies) was incubated with 5 μg anti-CSDE1 rabbit pAb (Novus Biologicals), anti-PABP1 rabbit pAb (Abcam) or anti-eIF4G rabbit pAb (Abcam) in 200 μl PBS-T (0.01% Tween) for 1 hr at room temperature. The beads were then blocked overnight in 1 ml 0.1% BSA in PBS on a rotating shaker at 4 °C. The beads were washed twice with PBS-T, and then equilibrated with 900 μl NT2 buffer (50 mM Tris-HCl (pH 7.4), 150 mM NaCl, 1 mM MgCl_2_, 0.05% NP40) supplemented with 400 μM VRC, 1 mM DTT and 20 mM final concentration of EDTA. The cell lysate was thawed on ice, centrifuged at 15,000 × g for 15 min to clear lysate of large particles. The cleared supernatant was transferred to microfuge tubes and stored on ice. Where indicated, 0.1 mg/ml RNase A or 10 U RNase ONE (Promega) was added to the lysate and incubated at room temperature for 30 min prior to IP. 100 μl of the cleared lysate was added to pre-equilibrated beads and incubated for 4 hr at 4 °C on a rotating shaker. The beads were pelleted on a magnetic stand, and washed 4–5 times with cold NT2 buffer. The beads were then boiled in SDS protein sample buffer and run on a 10% SDS-polyacrylamide gel, the proteins transferred to a nitrocellulose membrane (Amersham, GE Healthcare), blocked in 5% dried milk in TBS-T, and probed with primary (1:1000) and secondary antibodies (1:10,000). The protein bands were detected using Pierce^®^ ECL Western Blotting Substrate (Thermo Scientific).

### Metabolic labelling

Metabolic labelling of HeLa or U2OS cells was carried out in 6-well plates 48 hours after transfection with siRNA against Unr or a negative control siRNA. To deplete the cells of methionine, the cells were incubated in methionine-free media (Gibco, Thermo Fisher Scientific) for 2 hours. Labelling of nascent proteins was then carried out for 30 minutes using 100 μCi of EasyTag ^35^S-methionine (Perkin Elmer) per well. The cells were then washed in PBS and harvested in 1× Passive Lysis Buffer (Promega). The protein concentration of cell lysate was quantified by BCA protein assay (Pierce), and equal amounts of protein were boiled in SDS protein sample buffer and run on a 10% SDS-polyacrylamide gel. The gel was fixed, dried, and exposed to BioMax MR film (Kodak). ^35^S-labelled proteins were detected by autoradiography.

## Additional Information

**How to cite this article**: Ray, S. and Anderson, E. C. Stimulation of translation by human Unr requires cold shock domains 2 and 4, and correlates with poly(A) binding protein interaction. *Sci. Rep.* **6**, 22461; doi: 10.1038/srep22461 (2016).
